# Role of extracellular vesicles in the development of sepsis-induced coagulopathy

**DOI:** 10.1186/s40560-018-0340-6

**Published:** 2018-10-19

**Authors:** Toshiaki Iba, Hiroshi Ogura

**Affiliations:** 1 Department of Emergency and Disaster Medicine, Juntendo University Graduate School of Medicine, 2-1-1 Hongo Bunkyo-ku, Tokyo, 113-8421 Japan; 2 Department of Traumatology and Acute Critical Medicine, Osaka University Graduate School of Medicine, Osaka, Japan

**Keywords:** Extracellular vesicle, Exosome, Microvesicle, Sepsis, Coagulopathy

## Abstract

**Background:**

The advances of research on extracellular vesicles (EVs) are of particular interest to the clinicians as well as the researchers who are studying coagulation disorder in sepsis. Here, we intend to update the latest knowledge and currently unsolved problems that should be addressed.

**Main body:**

Secreted membrane-enclosed vesicles including apoptotic bodies, exosomes, ectosomes, microvesicles, and microparticles are generically called EVs. Though the basic structure of these vesicles is the same, i.e., originating from the plasma membrane, their characteristics differ significantly depending on their surface structures and interior components. Numerous studies have shown elevated levels of circulating EVs that exhibit proinflammatory and procoagulant properties during sepsis. These EVs are known to play important roles in the development of coagulation disorder and organ dysfunction in sepsis. Coagulation disorder in sepsis is characterized by activated coagulation, disrupted anticoagulant systems, and imbalanced fibrinolytic systems. These processes collaborate with one another and contribute to the development of disseminated intravascular coagulation (DIC), with devastating consequences. As part of this pathogenesis, the membrane-exposed tissue factor, phosphatidylserine and bioactive substances contained within the vesicles, such as histones, nucleosomes, and high-mobility group box 1, contribute to the development of DIC. EVs not only upregulate the procoagulant systems by themselves, but they also disseminate prothrombotic activities by transferring their procoagulant properties to distant target cells. Though the basic concept behind the role of procoagulant properties, EVs in the development of sepsis-induced coagulopathy has started to be unveiled, knowledge of the actual status is far from satisfactory, mainly because of the lack of standardized assay procedures. Recent advances and current problems that remain to be resolved are introduced in this review.

**Conclusion:**

The recent studies succeeded to elucidate the important roles of EVs in the progress of coagulation disorder in sepsis. However, further harmonization in terminology, methodology, and evaluation methods is required for future studies.

## Background

**Fig. 1:**
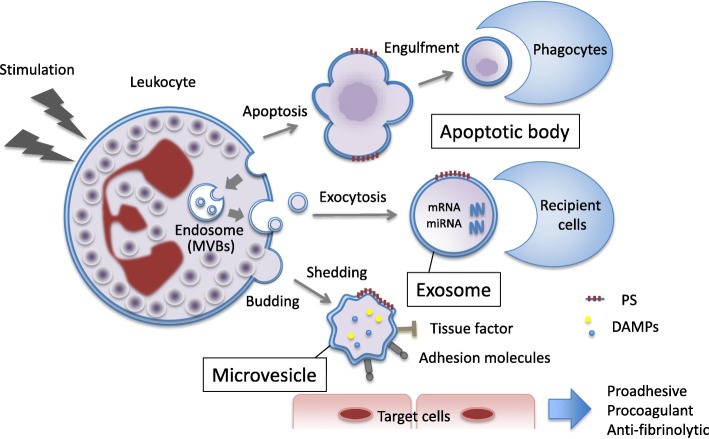
Different types of extracellular vesicles. Leukocytes can produce extracellular vesicles in response to certain stimuli. Apoptotic bodies are the final form of apoptotic cell-death and are known to be engulfed by phagocytes. Exosomes are secreted after multivesicular bodies (MVBs) fuse with the plasma membrane. Exosomes contain messenger RNA and micro RNA and are released by exocytosis. Microvesicles that express tissue factor and adhesion molecules and that carry damage-associated molecular patterns are shed from leukocytes. Extracellular vesicles present procoagulant properties expressed by phosphatidylserine on their surfaces. PS phosphatidylserine, DAMPs damage-associated molecular patterns

**Fig. 2:**
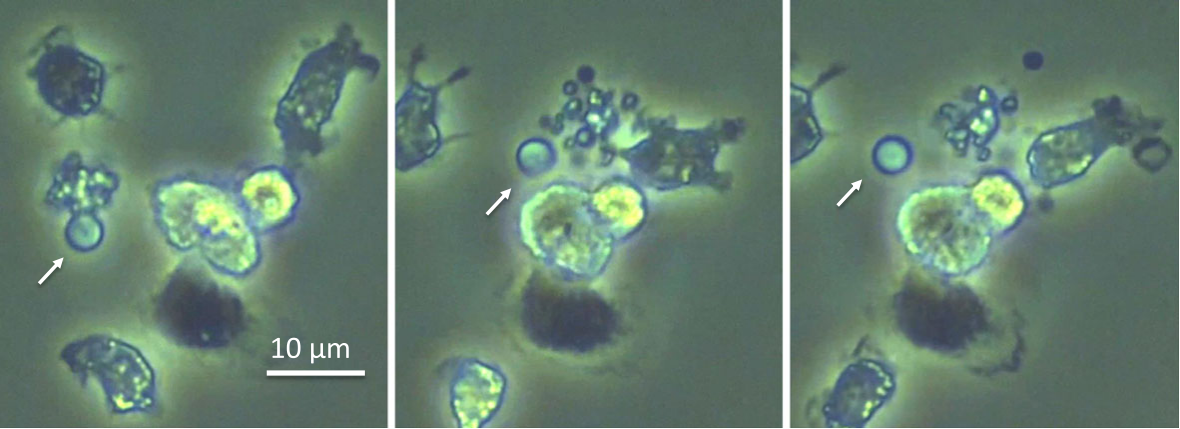
Shedding of extracellular vesicles. Extracellular vesicles are shed from the plasma membrane of apoptotic cells. Leukocytes were stimulated by lipopolysaccharide. Forty hours later, a 3-μm extracellular vesicle (white arrow) was released from the apoptotic body

For many years, extracellular vesicles (EVs) were considered to be “garbage bags” or debris, and it is true that EVs are used as “shipping containers” for cellular waste. For instance, the nucleus of the erythrocyte is expelled by this system during the maturation process. However, EVs are now considered to be important messengers in inflammatory signaling via cell-to-cell communication [1, 2], and they have been recognized as strong promoters of coagulation in sepsis [3, 4]. EVs are small (0.03–5.00  μm), spherical particles enclosed by bilayer phospholipid membranes. They can be released from the surface of almost any cell type into a variety of bodily fluids including plasma, saliva, cerebrospinal fluid, breast milk, semen, and urine. There are several sub-classes of EVs, including apoptotic bodies, exosomes, and microvesicles (formerly called microparticles), depending on their biogenesis and phenotypic origin (Table 1, Fig. 1) [5, 6]. Cells undergoing apoptosis release relatively large EVs with diameters of 0.5–5.00  μm that are referred to as apoptotic bodies (Fig. 2) [7]. Cells can also produce a more heterogeneous population of EVs with submicron diameters called exosomes and microvesicles [5].

**Table 1:** Extracellular vesicles

	Apoptotic body	Exosome	Microvesicle
Membrane	Plasma membrane	Endosome membrane	Plasma membrane
Size	0.5–5 μm	0.03–0.15 μm	0.1–5 μm
Biogenesis	Cellular disassembly/fragmentation	Endocytosis→exocytosis	Budding→shedding of plasma membrane
Functions	Suppression in inflammation	Cell-to-cell communication	Cell-to-cell communication
Surface markers	Phosphatidylserine	CD63, CD81, CD9, etc.	Adhesion molecules, tissue factor
Contents	Fragmented DNA, organelle	mRNA, miRNA	DAMPs (histones, HMGB1, etc.), proteases (MMP, CK18), etc.
Density	Unknown	1.10–1.14 g/mL	1.12–1.20 g/mL

*mRNA* messenger RNA, *miRNA* micro RNA, *DAMP* damage-associated molecular pattern, *HMGB1* high-mobility group box 1, *MMP* matrix metalloproteinase, *CK18* cytokeratin 18

With their ability to deliver proteins, lipids, and nucleotides from one cell to another, EVs have begun to attract attention in various different medical fields including immunology [8], cancer research [9, 10], cardiovascular diseases [11], inflammatory diseases [12], and autoimmune diseases [13]. EVs can also spread the characteristics of their parent cell by transferring receptors, organelles, messenger RNA, micro RNA, and other proteins to distant cells [14]. Procoagulant EVs have been reported to play significant roles in the activation of coagulation during sepsis [3, 4, 15]. These procoagulant properties are primarily based on the presence of tissue factor (a major initiator of the coagulation cascade) and phosphatidylserine (a procoagulant phospholipid that supports the assembly of blood-clotting enzyme complexes). Earlier studies focused on the roles of the most common EVs, specifically platelet-derived EVs, in the activation of coagulation [16]. Although platelet-derived EVs are dominant in sepsis-induced coagulopathy, EVs from other cell types including leukocytes, endothelial cells, and red blood cells also contribute to pro- and anti-inflammatory reactions during sepsis [17]. In this review, we have focused on the roles of various types of EVs originating from different cells in the development of coagulation disorder in sepsis.

## Main text

### Classification and generation

EV is a generic name describing membranous cellular fragments including apoptotic bodies, exosomes, microvesicles, microparticles, ectosomes, and other subsets [18]. The terminology is still confused, for example, microvesicle is almost the same as microparticles. Microvesicles were initially characterized by their procoagulant activity and called as microparticles. EVs can originate from all cell types and are released into various body fluids [5, 6]. Though some confusion regarding their classification remains, all EVs are comprised of membranous proteins, phospholipids, and other molecules that originate from the parental plasma membrane and include intracellular components such as proteins and RNAs. The International Society for Thrombosis and Haemostasis (ISTH) and the International Society for Extracellular Vesicles (ISEV) have collaboratively discussed the nomenclature for EVs, the presence of EVs in fluids, methods of isolation and detection, and emerging clinical implications [19].

**Fig. 3:**
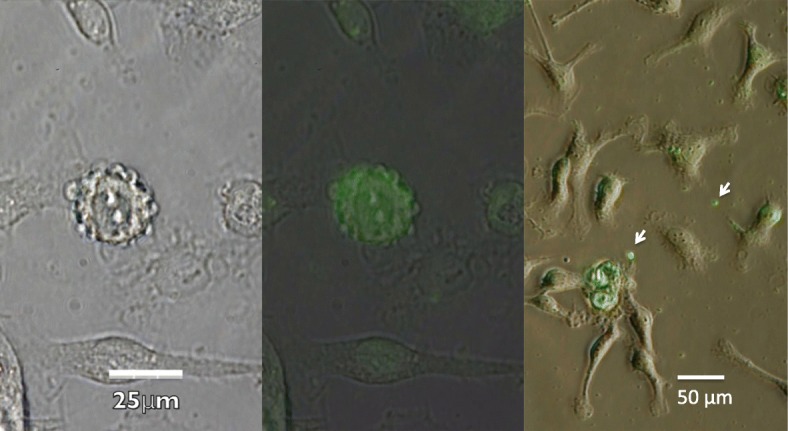
Budding of extracellular vesicle. Bright view of the budding leukocyte (left). The leukocyte was stained with anti-CD11b antibody conjugated with green fluorescent protein (middle). Particles apart from the cells were stained with anti-CD11b antibody conjugated with green fluorescent protein (right)

**Fig. 4:**
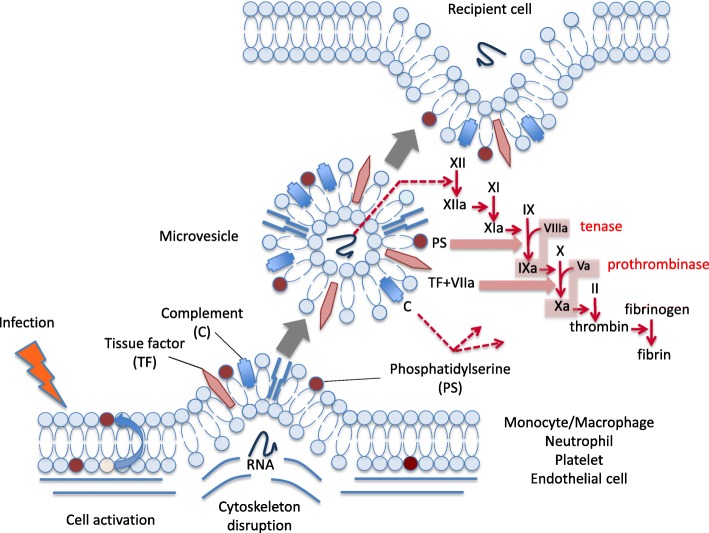
Procoagulant activities of the extracellular vesicles. Monocytes and neutrophils play major roles in the activation in coagulation during sepsis. They express tissue factor on the cellular surface that initiates extrinsic pathway and also express phosphatidylserine that triggers intrinsic pathway of the coagulation cascades on the outer-envelope of the plasma membrane. Tissue factor and phosphatidylserine are also expressed on the surface of microvesicles released from the other parent cells such as platelets and endothelial cells

Different types of EVs are generated by individual mechanisms of biogenesis [5] (Fig. 1). For example, infection-induced apoptosis results in the formation of apoptotic bodies that modulate inflammatory and immune responses [20] (Fig. 2). Meanwhile, the inward budding of the cytoplasmic membrane is the first step in the generation of exosomes, which then proceeds to the formation of endosomes (multivesicular bodies [MVBs]) and ultimately to exocytosis via fusion with the plasma membrane [5]. In contrast, microvesicles are generated by the outward budding of the plasma membrane and are released via shedding [21] (Figs. 3 and 4). Certainly, these individual mechanisms cannot always be divided clearly. For instance, microvesicles are also known to be formed during the course of apoptosis. Though cells have been shown to generate microvesicles after activation and during the course of apoptosis, these structures are theoretically distinct in their compositions and sizes from other subcellular structures. However, since the structures and compositions change in response to different stimuli, their actual classification is rather difficult [22] and efforts to address this issue have not yet been successful [21]. On the other hand, irrespective of their dimensions, origins, and release processes, the characteristics of EVs are defined by their components. Thus, it is more meaningful to divide EVs depending on their phospholipid bilayer, membrane-bound or transmembrane proteins, and endoplasmic cargo molecules. Regardless of the classification difficulties that presently exist, it is more important to elucidate the roles of EVs as messengers, their significance in pathogenesis, and their usefulness as biomarkers.

### Membranous and membrane-binding procoagulant factors

#### Tissue factor

There are some excellent review papers that explain the connection between EVs and sepsis-induced coagulopathy [23]. EVs convey pro-coagulative tissue factor and phosphatidylserine on their surfaces, thereby modulating the inflammatory response and coagulation [24, 25] (Fig. 4). Tissue factor is a transmembrane receptor for Factor VII/VIIa that plays a principal role in the initiation of the extrinsic coagulation pathway. It is constitutively expressed by extravascular cells for prompt hemostasis, while none of the cells present in the circulating blood express tissue factor under physiological conditions. Conversely, tissue factor is presented within the blood during sepsis for the compartmentalization of microorganisms. Thus, this reaction is thought to be part of the host defense mechanisms in response to infection [26]. The problem is that the excessive activation of coagulation induces tissue malcirculation, leading to organ dysfunction. Consequently, genetically altered mice with either low tissue factor expression [27] or low Factor VII levels [28] exhibit improved survival after endotoxin treatment, compared with wild-type mice.

Tissue factor is detected primarily in the cellular fraction containing monocytes. Activated monocytes upregulate tissue factor expression on their surfaces, and EVs conveying tissue factor are thought to be released predominantly from activated monocytes [29]. Currently, tissue factor is known to be expressed by other cells, such as endothelial cells and platelets, as well as by circulating EVs from cells involved in sepsis, and the activity of procoagulant tissue factor on EVs is reportedly associated with the severity of sepsis [30, 31]. Although EV-associated tissue factor represents a small fraction of the total tissue factor component in blood, its presence has been reported to be associated with the development of disseminated intravascular coagulation (DIC) [32]. But the fact is not simple, and Matsumoto et al. [33] reported that the numbers of anticoagulant EVs such as endothelial protein C receptor-positive EVs and thrombomodulin-positive EVs elevated together with the increase of tissue factor-positive EVs.

#### Phosphatidylserine

Phosphatidylserine exposed on EVs from platelets, leukocytes, and endothelial cells contributes to the procoagulant activity during sepsis. A landmark study in this area was performed by Dvorak et al. [34]. They showed that several cancer cell lines expressed coagulant activities associated with membrane vesicles. They postulated that these vesicles provided a phospholipid surface enabling the assembly of tenase (Factor VIIIa/Factor IXa) and prothrombinase (Factor Va/Factor Xa) complexes and exhibited a thromboplastin-like activity. Zhang et al. [35] reported that phosphatidylserine exposed on blood cells was markedly higher in septic patients and that endothelial cells treated with EV-containing serum obtained from septic patients exhibited more exposed phosphatidylserine than those treated with serum from healthy controls. Their observation demonstrated that the procoagulant properties of effector cells can be transmitted to target cells by EVs. In this regard, Tripisciano et al. [36] performed an interesting study. They added platelet-derived EVs to vesicle-free human plasma and induced thrombin generation; this phenomenon was then efficiently inhibited by annexin V, which binds and masks phosphatidylserine, but not by anti-tissue factor antibodies, indicating that the thrombin generation was primarily due to the exposure of phosphatidylserine on EVs. Additionally, phosphatidylserine is known to represent a platform for thrombin generation through the activation of the complement system [37].

### Intra- and extracellular procoagulant and immune modulatory factors

#### Neutrophil extracellular traps

Sepsis is associated with a massive release of leukocyte-derived EVs, particularly neutrophil-derived EVs that retain the DNA, histones, and other components of the mother neutrophils, contributing to the pro-coagulant activity [38]. Gould et al. [39] demonstrated that neutrophil extracellular traps (NETs), a mesh-like component of DNA fibers comprised of histones and antimicrobial peptides, have a massive ability to activate the coagulation cascade, preventing bacterial dissemination [40]. Histones are leaked out from the dead cells as components of nucleosomes or expelled from neutrophils in the active process of NETs formation. Nair et al. [41] reported that the presence of vesicle-associated histones in the lipopolysaccharide-challenged mice. They showed that actively released histones from leukocytes locate on the outer surface of vesicles and contribute to both inflammation and coagulation. Accordingly, DIC is associated with the profound generation of leukocyte-derived EVs, especially neutrophil-derived EVs that holds the same components as NETs [42]. Delabranche et al. [43] recently noted the presence of NETs as identified by myeloperoxidase-DNA complexes and nucleosomes in sepsis-induced DIC. Hence, NET components containing EVs are likely to be involved in the upregulation of the coagulation system during sepsis-induced coagulopathy [44, 45].

#### Damage-associated molecular patterns (DAMPs)

In sepsis, activated or damaged cells release various cellular components such as damage-associated molecular patterns (DAMPs), NETs, and EVs into the extracellular space, and these components play key roles in the innate immune system and in tissue repair [46, 47]. At the same time, they are involved in the pathogenesis of systemic inflammation and thrombogenesis, which can directly lead to organ dysfunction [46, 48]. EVs are known to transfer DAMPs (e.g., histones, chromosomal DNA, nucleosome, mitochondrial DNA, high-mobility group box 1 protein [HMGB1], and heat shock protein [HSP]) inside cells as well as on the cell surface [49]. Platelet-derived EVs constitute the majority of circulating EVs and are preferentially associated with granulocytes and monocytes, while they rarely interact with lymphocytes. Further flow cytometric differentiation of monocyte subsets has provided clear indications for the preferential association of platelet-derived EVs with intermediate (CD14^++^CD16^+^) monocytes in whole blood [50].

Similar to NETs, DAMPs in EVs are known to contribute to an increased prothrombotic reaction and DIC in sepsis [46]. Leukocytes and endothelial cells are involved in the pathophysiology of sepsis from an early stage, whereas the involvement of activated platelets is delayed. Regarding the initiator of coagulation in sepsis, Delabranche et al. [51] reported that leukocyte-derived EVs and endothelial cell-derived EVs are more highly elevated in plasma in DIC at earlier stages, while the elevation of the soluble glycoprotein V, a marker released from the platelet surface depending on the thrombin/platelet ratio, is delayed. Thus, leukocyte-derived and endothelial cell-derived EVs are thought to be initial players, while platelet-derived EVs are thought to have subsequent roles [52].

#### Complements

Though the dark-side of EVs has been closed up, EVs also play roles in immunomodulation. EVs are involved in a variety of cellular processes including innate immune response, tissue metabolism, and coagulation, and these factors altogether regulate the inflammatory reactions. Complement component 5a (C5a), an anaphylatoxin, is known to contribute to the coagulopathy and organ failure in sepsis. The inhibition of C5a is reported to attenuate the consumptive coagulopathy and subsequent organ failure in the baboon model of sepsis [53]. Importantly, EVs not only propagate the inflammation but they also possess anti-inflammatory properties and contribute to the immunomodulation. For example, complement factors and complement regulators locate in or on the surface of EVs and modulate inflammation and cellular damage. Thus, EVs are recognized as the regulator of complement activity and contributes to balancing the pro- and anti-inflammatory immune response [54]. However, the molecular mechanisms behind this interaction remain elusive and require further investigation.

#### Messenger RNA and micro RNA

Exosomes contain messenger RNAs and micro RNAs. RNAs are known as major DAMPs, and RNAs in EVs can stimulate multiple innate immune signaling pathways and coagulation cascades that lead to thrombotic complications and organ dysfunction. Micro RNAs are noncoding RNA molecules that emerge as important regulators of every single cellular response to the infection. Micro RNAs induce either messenger RNA degradation or translational repression by binding to the specific sequence motifs within the 3′ untranslated region. In septic patients, increased levels of exosomes containing potent micro RNAs and micro RNA-clusters have been reported along with the upregulated immune system [55]; however, the further detail in the regulation of gene expression of these RNAs and their relation to the coagulation system have not been unveiled yet.

### Sources

#### Leukocytes

Leukocytes, especially neutrophils, are known to play key roles in the development of sepsis-associated DIC [42, 56, 57]. Delabranche et al. [42, 43] reported that the leukocyte-derived EV count increases significantly during sepsis and that this count can be used as an early and relevant biomarker of sepsis-associated DIC in humans. They also reported that the leucocyte-derived CD11a^+^-EVs/leucocyte and neutrophil-derived CD66b^+^-EVs/neutrophil count ratios were significantly higher in DIC patients, compared with those in non-DIC patients [42]; these findings agree with the observed increase in endothelial-derived EVs [42]. Taken together, reported results suggest that sepsis-associated DIC results from endothelial injury after an inflammatory burst induced by the recruitment of leucocyte-derived EVs.

Leukocyte-derived EVs that originate from neutrophils, monocytes/macrophages, and lymphocytes disrupt vascular homeostasis via their cytoplasmic contents, such as reactive oxygen species (ROS), nucleotides, nucleic proteins, proteolytic enzymes, and HMGB1 [58]. A sepsis model was reproduced by the treatment with EVs from septic rats. This experimental model of sepsis exhibited an increase in superoxide production and nuclear factor-κB activity, an increase in inducible nitric oxide synthase (inducing nitric oxide overproduction), and a decrease in constitutive endothelial nitric oxide synthase activation [59]. The leukocyte-derived EVs also play key roles in the activation in thrombogenesis, and tissue-factor-bearing EVs were associated with the procoagulant activity [60, 61]. Of note, tissue factor-bearing EVs can interact with leukocytes by paracrine transfer [62]. Interestingly, thrombomodulin and tissue factor coexist on the surface of monocyte-derived EVs in the resting state; when the monocytes are stimulated by lipopolysaccharide, however, the tissue factor activity becomes dominant on EVs [63]. Furthermore, monocyte-derived EVs can induce endothelial cell apoptosis, resulting in the loss of the anticoagulant properties of the vascular luminal surface [64].

#### Platelets

Platelet-derived EVs are very important for hemostasis, and platelets themselves can be considered as EVs released from megakaryocytes [11]. Platelets are involved in a variety of pathophysiological responses including the host-defense response against infection [65], inflammation, angiogenesis, and tissue regeneration [66]. Recent studies have revealed a number of significant roles of platelet-derived EVs in these responses. Indeed, platelet-derived EVs represent the majority of EVs circulating in the blood [67]. In septic patients, procoagulant EVs are mainly released by platelets [68] and supplemented by endothelial cells, neutrophils, and monocytes [69]. It is noteworthy that together with the up-regulation of tissue factor on these parent cells, blood-borne tissue factor-positive EVs are also co-responsible for the prothrombotic milieu that underlies DIC [70]. Lehner et al. [71] reported that the endothelial-derived EV count does not increase significantly even when the patient is in shock. In contrast, the count of EVs released from activated platelets is remarkably elevated and is correlated with mortality.

Platelet-derived EVs contain unique subsets of proteins derived from parent cells; in recent years, it has become clear that these EVs have essential biological functions. They participate in blood coagulation by providing a source of tissue factor as well as negatively charged phosphatidylserine, creating a stage where clotting factor complexes can assemble [72]. A lipopolysaccharide-induced model of peritonitis showed an increase in platelet-derived procoagulant EVs and coagulation disorder from an early stage of sepsis [73]. Furthermore, Ohuchi et al. [74] reported associations between the platelet-derived EV/platelet ratio and both hospital mortality and DIC in critically ill patients.

Regarding the origin of tissue factor, mononuclear phagocytes and endothelial cells have been intensively studied and reported to induce the synthesis during sepsis [75]. In the clinical study, tissue factor expression was observed in monocytes from patients with infection [76]. Meanwhile, Darbousset et al. [77] reported that neutrophils are the main source of blood-borne tissue factor, however, other studies have reported that neutrophils do not synthesize tissue factor but can acquire tissue factor by gaining monocyte/platelet-derived microvesicles [78]. Thus, further investigation is required to clarify the major cellular source of septic coagulopathy.

#### Endothelial cells

Endothelial cells are the frontline players in the maintenance of the antithrombogenicity of blood vessels. They produce antithrombotic proteins, lipids, and gas and express glycocalyx on their surfaces. However, inflammatory stimuli induce endothelial cell death, and apoptotic endothelial cells are postulated to play some roles in the activation of coagulation [79]. Endothelial cells lose their antithrombotic properties and begin to act in an opposite manner, detaching from the basement membrane and undergoing rapid clearance from the circulation [80]. Circulating endothelial cells have been hypothesized to be an indicator of endothelial cell damage, and some studies have reported an increase in circulating endothelial cells under septic conditions [81, 82]. However, whether these circulating endothelial cells have a linear correlation with procoagulant activity in sepsis remains unknown.

Numerous studies have reported that endothelial cell-derived EVs are associated with DIC [40, 51]. Interestingly, activated endothelial cells reportedly increase their procoagulant activity during sepsis by enhancing the production of EVs that bind to neutrophils. Ogura et al. [83] reported that endothelial-derived EVs stimulate the oxidative activity of neutrophils and induce the activation of coagulation in sepsis. In addition to the aforementioned mechanisms, the exposure of complement proteins C5b-9 to endothelial cells stimulates EV formation with the expression of Factor Va binding sites and prothrombinase activity [84].

The complexity is further revealed by the fact that endothelial cell-derived EVs present not only procoagulant surface antigens, but also anticoagulant antigens such as thrombomodulin and endothelial protein C receptor [33]. Furthermore, the procoagulant/anticoagulant balance seems to change depending on the conditions; thus, the role of endothelial cell-derived EVs in the progression of sepsis-induced coagulopathy remains uncertain.

#### Erythrocytes

Erythrocyte-derived EVs are known to express phosphatidylserine on their membrane surfaces, but the significance of this procoagulant activity remains controversial. In the case of blood transfusion, erythrocyte-derived EVs reportedly increase during the storage of blood units, and patients who receive transfusions can suffer posttransfusion hemostatic complications [85]. Thalassemia patients are known to have a high level of circulating erythrocyte-derived and platelet-derived EVs. Agouti et al. [86] reported that the phospholipid-dependent procoagulant activity was correlated with platelet-derived EVs but not with erythrocyte-derived EVs. A similar observation was also recognized in immune thrombocytopenic purpura [87]. Koshiar et al. [88] explained that the erythrocyte-derived microparticle surface is suitable for the anticoagulant reactions of protein C and that this may help to balance the coagulation status. Since red blood cells are easily damaged in patients with sepsis-induced coagulopathy and patients have elevated levels of circulating erythrocyte-derived EVs, these EVs may play some roles in activated thrombin generation during sepsis.

### Detection and assay procedures

#### Pre-analytical steps

Pre-analytical steps, such as handling, storage, and centrifugation, have major impacts on the variability of EV analyses [89]. Numbers of assays and phenotyping methods coexist but are not necessarily comparable, making the interpretation of results across studies difficult [90]. The procedures for centrifugation, freeze-thaw cycles, time delays between blood collection and plasma preparation, and storage should ideally be uniform for all studies. The density gradient used for ultracentrifugation, in particular, differs among approaches for isolating EVs [91, 92], and the isolated EVs are significantly influenced by conditions such as the applied gradients and osmotic pressure [93]. Though a consensus for a standardized procedure for EV measurements does not yet exist, the ISTH has provided technical protocols and recommendations for the detection and measurement of EVs [94]. Nevertheless, further validation and revision of the current method have not yet been performed.

#### Flow cytometry

Circulating EVs can be analyzed using a number of different techniques. Among them, flow cytometry is probably the most commonly used technique for the investigation of EVs. A flow cytometer guides cells and smaller particles including EVs through a laser beam in a hydrodynamically focused fluid stream. The front detector is placed in line with the laser beam and measures the forward-scattered light (FSC), while the other detector measures the side-scattered light (SSC) perpendicular to the beam. Based on light-scattering theory, larger particles, such as cells and apoptotic bodies, predominantly scatter the light in a forward direction. Hence, FSC is associated with particle size. Particles that are smaller than the light wavelength, such as organelles and internal vesicles, scatter more light in a perpendicular direction; thus, SSC is associated with the complexity of the cellular structure. Flow cytometric analysis allows certain cell types to be distinguished from one another, but the detection limit remains insufficient (approximately 0.2–0.4 μm, depending on the equipment) [95, 96].

In fluorescence flow cytometry, the fluorescence from a single particle present in a hydrodynamically focused fluid stream is measured. Therefore, fluorescence-activated cells can be distinguished from other vesicles based on the spectral properties of the fluorescence signal using this technique [97]. The cellular origin of each EV can be determined by assessing the antigens exposed on the surface. Typical antigens are CD11b for leukocytes, CD14 for monocytes, CD41 for platelets, CD105 for endothelial cells, CD235a for erythrocyte, and CD142 for tissue factor (Table 2). However, the complete discrimination of EVs based on the detection of surface markers remains difficult because of the lack of specific antibodies. Moreover, hundreds of various proteins have been reported, and the cellular phenotypes change according to cellular responses to stimuli.

**Table 2:** Surface antigens

Origin	Antigen (CD)	Alternative
Platelet	CD41	GP IIb (integrin αIIb)
	CD42a	GP IX
	CD42b	GP Ib
	CD42d	GP V
	CD61	GP IIIa (integrin β)
	CD62P	P-selectin
	CD63	LIMP
Leukocyte	CD11a	LFA-1 (integrin αL)
	CD11b	MAC-1 (integrin αM)
	CD13	APN (Aminopeptidase-N)
	CD14	LPS-R (lipopolysaccharide receptor)
	CD16	Fc receptor FcγRIII
	CD66b	CEA antigen-like subfamily
Endothelial cell	CD31	PECAM-1
	CD51	Vitronectin receptor
	CD54	ICAM-1
	CD62E	E-selectin
	CD105	Endoglin
	CD144	VE-cadherin
	CD146	Mel-CAM
Erythrocyte	CD235a	Glycophorin-A
	CD238	Metallopoptidase
Tissue factor	CD142	Thromboplastin

*CD* cluster of differentiation, *GP* glycoprotein, *LIMP* lysosomal integral membrane protein, *LFA* lymphocyte function-associated antigen, *MAC* macrophage, *CEA* carcinoembryonic antigen, *PECAM* platelet endothelial cell adhesion molecule, *ICAM* intracellular adhesion molecule, *Mel-CAM* melanoma cell adhesion molecule

Using this procedure, correlations between circulating EVs and disease status have been detected for various inflammatory diseases, including sepsis [40, 51]. EVs are fascinating candidates for novel biomarkers, but their clinical relevance is hampered by methodological concerns and a lack of standardized procedures. In addition, considerable differences in the sensitivity of flow cytometry exist, and the background detection of EVs using different devices has been shown to exhibit a poor interlaboratory comparability. Accordingly, the ISTH has released a standard procedure to reduce inter-institutional differences and has distributed polystyrene beads to calibrate a scatter-based diameter gate to improve comparability [94].

#### Functional assays

The procoagulant properties of EVs can be measured using a functional assay, namely the procoagulant activity assay. This assay measures the activity of tissue factor based on its ability to activate Factor X. Mooberry et al. [98] demonstrated that EVs isolated from plasma following endotoxin treatment exhibit increased tissue factor activity using this procoagulant assay. They reported that EVs added to re-calcified platelet-poor plasma decreased the clotting time and shortened the lag time and the time to peak using calibrated automated thrombography. Thrombin generation measured using a procoagulant activity assay and flow cytometry for phosphatidylserine and tissue factor-expressing EV are also reportedly well correlated [99].

Another functional assay is the prothrombinase activity assay. This assay is basically an assay for Factor Xa and prothrombin inhibitors; for this application, however, the thrombogenicity of the membrane is detected. The prothrombinase complex, which includes Factors Va, Xa, calcium, and prothrombin, is required for thrombin formation, and phospholipids offer binding sites for this reaction. EVs have a phosphatidylserine-rich procoagulant membrane that can accelerate prothrombinase activity [100]. Shaver et al. [101] reported that elevated levels of circulating platelet EVs are independently associated with a reduced risk of acute respiratory distress syndrome in critically ill patients. Whether this effect is due to the effects of EVs on systemic coagulation remains uncertain.

#### Other analytic approaches

There are many other analytic approaches including western blots, global proteomic analyses using mass spectrometry techniques, and enzyme-linked immunosorbent assays (ELISAs). Among them, ELISAs are both easy to perform and reproducible [102, 103]; however, the major problem associated with this assay is the lack of an ideal isolation technique. In the case of examining EVs in plasma, the bulk of the sample will contain soluble proteins, HDL, and LDL that may affect the result. The samples also contain soluble glycoproteins such as the GPIb/IX/V complex, which will also react with the antibody used in the analysis. Thus, the results of ELISAs should be interpreted with caution.

For the same reason, the composition of EVs varies significantly depending on the protocols used when polymer-based methods are applied to precipitate EVs. These methods do not exclusively isolate EVs and are likely to co-isolate other molecules such as RNA-protein complexes. Therefore, standardized research procedures are required for further EV research [18].

### Therapeutic target

The significance of EVs as a therapeutic target remains to be elucidated. Since EVs are a major contributor to the evolution of coagulation disorder in an individual, preventing their release could be beneficial under certain conditions. The potential efficacy of some anticoagulants for the management of EVs has been reported. Boisrame-Helms et al. [104] reported that treatment with activated protein C significantly reduced the generation of leucocyte-derived EVs, thereby limiting vascular inflammation and favoring hemodynamic improvement in a septic shock model.

Thrombomodulin is a transmembrane glycoprotein that is mostly expressed by endothelial cells and plays pivotal roles for the control of coagulation [105, 106]. Thrombin-thrombomodulin complex catalyzes the activation of protein C, leading to the inactivation of Factor Va and Factor VIIIa. In addition, the lectin-like domain of thrombomodulin has been shown to express anti-inflammatory properties by inhibiting leukocyte adhesion to the endothelium and degrading proinflammatory DAMPs, such as HMGB1 and histones [107, 108]. Recombinant thrombomodulin is expected to suppress over-activated coagulation, to decrease leukocyte activation, and to attenuate subsequent consecutive inflammatory processes in sepsis [109]. In a related report, Helmes et al. [110] reported that recombinant thrombomodulin limited the release of leukocyte-derived EVs and decreased the expression of procoagulant phospholipids on the EV surface.

Other than the anticoagulants, Essandoh et al. [111] reported the blocking of EVs release from bacteria-infected macrophages by pre-treatment with a sphingomyelinase inhibitor in septic mice had cardioprotective effects and prolonged survival. As described before, almost all of the cells are capable of releasing EV and their roles have not been sufficiently investigated. This indicates that they certainly modulate septic processes by controlling the activation and proliferation of the immune cells; however, since the existing evidence strongly suggests that EVs have both protective and detrimental roles, whether the suppression of EVs function can induce beneficial effects to the host is still unknown.

### Future perspectives

In the last two decades, significant progress has been achieved in identifying the roles of EVs in sepsis. However, the mutual interactions between EV release and coagulation disorder have not been fully elucidated at the present time. Several promising speculations pertaining to the roles of EVs in sepsis-induced coagulopathy have been made, and further advances are expected. However, more precise and standardized means of defining and characterizing EVs are needed. At present, the sizes of EVs have not been clearly defined, and the terms “exosome” and “microvesicle” often encompass mixtures of heterogeneous populations of EVs. The lack of established definitions and characterization results has led to inconsistencies in study results. Thus, the standardization of terminology and assay procedures are urgent matters. Since 2014, the ISTH and the ISEV have collaborated to discuss these issues. Further progress in unification is expected.

## Conclusions

Sepsis-induced coagulopathy is a typical example of the tight connection between inflammation and thrombosis. Numerous studies have shown that a variety of proinflammatory agents, such as cytokines, DAMPs, ROS, complements, and other humoral mediators, bridge these two reactions. Recently, EVs have been identified as new contributors that play key roles in this field. EVs directly and collaboratively activate coagulation systems that lead to the further upregulation of inflammation and life-threatening organ dysfunction and DIC. EVs are known to be responsible for the secretion, exchange, and transmission of important active biomolecules in sepsis. Indeed, EVs represent an essential mechanism in intercellular communication, and the roles of EVs in infection and thrombosis have been increasingly recognized. However, research on EVs has just begun, and this field of study remains chaotic. Standardized approaches to fundamental studies are urgently needed.

## Abbreviations

DAMP: Damage-associated molecular patterns
DIC: Disseminated intravascular coagulation
ELISA: Enzyme-linked immunosorbent assay
EV: Extracellular vesicle
FSC: Forward-scattered light
ISEV: International Society on Extracellular Vesicles
ISTH: International Society for Thrombosis and Haemostasis
MVB: Multivesicular body
NET: Neutrophil extracellular traps
ROS: Reactive oxygen species
SSC: Side-scattered light
